# Study protocol for iQuit in Practice: a randomised controlled trial to assess the feasibility, acceptability and effectiveness of tailored web- and text-based facilitation of smoking cessation in primary care

**DOI:** 10.1186/1471-2458-13-324

**Published:** 2013-04-10

**Authors:** Stephen Sutton, Susan Smith, James Jamison, Sue Boase, Dan Mason, A Toby Prevost, James Brimicombe, Melanie Sloan, Hazel Gilbert, Felix Naughton

**Affiliations:** 1 Behavioural Science Group, Institute of Public Health, University of Cambridge, Cambridge, England, UK; 2 Primary Care Unit, Institute of Public Health, University of Cambridge, Cambridge, England, UK; 3 Department of Primary Care and Public Health Sciences, King’s College London, London, UK; 4 Department of Primary Care and Population Health, University College London, London, UK; 5 Institute of Public Health, University of Cambridge, Forvie Site, Robinson Way, Cambridge, CB2 0SR, UK

## Abstract

**Background:**

Primary care is an important setting for smoking cessation interventions. There is evidence for the effectiveness of tailored interventions for smoking cessation, and text messaging interventions for smoking cessation show promise. The intervention to be evaluated in this trial consists of two components: (1) a web-based program designed to be used by a practice nurse or other smoking cessation advisor (SCA); the program generates a cessation advice report that is highly tailored to relevant characteristics of the smoker; and (2) a three-month programme of automated tailored text messages sent to the smoker’s mobile phone. The objectives of the trial are to assess the acceptability and feasibility of the intervention and to estimate the short-term effectiveness of the intervention in increasing the quit rate compared with usual care alone.

**Methods/design:**

The design is a two parallel group randomised controlled trial (RCT). 600 smokers who want to quit will be recruited in up to 30 general practices in the East of England. During a consultation with an SCA, they will be individually randomised by computer program to usual care (Control) or to usual care plus the iQuit system (Intervention). At the four-week follow-up appointment, the SCA will record smoking status and measure carbon monoxide level. There will be two further follow-ups, at eight weeks and six months from randomisation date, by postal questionnaire sent from and returned to the study centre or by telephone interview conducted by a research interviewer. The primary outcome will be self-reported abstinence for at least two weeks at eight weeks. A sample size of 300 per group would give 80% power to detect an increase in quit rate from 20% to 30% (alpha = 0.05, 2-sided test). The main analyses of quit rates will be conducted on an intention-to-treat basis, making the usual assumption that participants lost to follow up are smoking.

**Discussion:**

This trial will focus on acceptability, feasibility and short-term effectiveness. The findings will be used to refine the intervention and to inform the decision to proceed to a pragmatic trial to estimate longer-term effectiveness and cost-effectiveness.

**Trial registration:**

ISRCTN56702353

## Background

The intervention to be evaluated in the iQuit in Practice trial consists of two components: (1) a web-based program designed to be used by a practice nurse or other smoking cessation advisor (SCA) in a consultation with a smoker; the program generates a cessation advice report that is highly tailored to relevant characteristics of the smoker; and (2) a three-month programme of automated tailored text messages sent to the smoker’s mobile phone. The iQuit system is a potentially cost-effective approach that is designed to enhance the effectiveness of the consultation, without requiring SCAs to radically change the way they advise and treat smokers and to provide continuing support to smokers during their quit attempt.

The following sections provide a justification for the choice of setting (primary care/general practice) and for the two components of the intervention (tailored advice reports and tailored text messaging).

### Primary care as a setting for smoking cessation interventions

Primary care, and general practice in particular, is an important setting for smoking cessation interventions. In the context of the National Health Service (NHS) Stop Smoking Services, more smokers are treated in primary care than in other settings [1]. Smokers can be identified from practice records and recruited proactively into smoking cessation programmes or trials of such interventions, for example by a letter from the general practitioner (GP) [2,3]. The guidelines on smoking cessation for health professionals in the UK endorse an important role for practice nurses in encouraging smokers to stop and providing advice and support [4]. A systematic review of nurse-delivered interventions for smoking cessation found them to significantly increase the likelihood of quitting compared with a control or usual care [5]. Current guidance from the National Institute for Health and Clinical Excellence (NICE) recommends a number of effective interventions for smoking cessation suitable for use in primary care, including brief interventions, individual behavioural counselling, pharmacotherapies and self-help materials [6]. A recent trend in the UK is for health care assistants (HCAs) to be given responsibility for delivering smoking cessation advice and support, but to date no studies have evaluated the effectiveness of interventions delivered by this group of health professionals.

### Tailored interventions for smoking cessation

Tailored interventions use data collected on or about an individual to make the information provided to the individual (e.g. smoking cessation advice) more personally relevant and therefore more likely to be attended to, read and understood, and acted upon. A Cochrane review of self-help interventions for smoking cessation [7] identified 25 trials of materials tailored to the characteristics of individual smokers, which overall showed a small benefit (N = 28,189; RR 1.31, 95% CI 1.20 to 1.42). The evidence was strongest for tailored materials compared to no intervention, but also suggested that tailored materials are more effective than standard materials.

A meta-analysis of computer-tailored interventions for health behaviour change identified 32 studies in which a group receiving a tailored intervention for smoking cessation was compared with a group receiving a non-tailored intervention or assessment only (it is not stated whether all these studies were randomised controlled trials) [8]. The mean effect size (Hedges g) based on self-reported point prevalence abstinence was 0.16 (95%CI 0.12 to 0.19, p < .001), which the authors interpret as a small-to-medium effect size; the mean quit rates at final follow-up were 20% in the tailored intervention group versus 14% in the comparison group. The effect size for the subset of 16 studies reporting prolonged abstinence outcomes (at 10 weeks, 6 months and 9 months) was 0.24 (95%CI 0.20 to 0.31, p < .001), which the authors interpret as a large effect size.

We have conducted three trials of tailored cessation advice delivered by letters or reports sent through the post [9,10] or via the web [11], with mixed results. Our first trial showed that tailored advice letters had a small but useful effect on quit rates at six months among those participants (callers to a quitline) who were cigarette smokers at baseline [9]. Our more recent trial of tailored advice reports, conducted in primary care, found an effect on quit attempts [10]; the findings for prolonged abstinence were consistent with there being a small positive effect of the intervention, though this was not statistically significant. In the third trial, tailored advice delivered via the web was not more effective than standardized (non-tailored) advice [11].

### Text messaging for smoking cessation

Text messaging (also called short message service or SMS) has become an increasingly important communication medium with 11 million text messages being sent an hour in the UK [12]. The technology enables short messages to be sent easily, quickly and inexpensively. In the UK, mobile phone ownership is high and texting is widely used. 92% of UK adults own a mobile phone [13]. In 2011, 58% of adults used text messages at least once a day to communicate with friends and family. The average person in the UK sent 200 SMS text messages per month in 2011 [13]. Mobile phone ownership varies by age (98% of 16–24 year olds; 68% of those aged 65+) [13] and by income (67% households in the lowest income decile group reported ownership in 2010, compared with 90% in the highest income decile group) [14]. Nevertheless, this medium provides a way of delivering smoking cessation advice and support to people across age and socioeconomic groups.

SMS is being used in a number of smoking cessation programmes [15,16]. Studies have shown that SMS-based smoking cessation programmes are generally feasible and acceptable [17], but data on effectiveness, particularly in the longer term, are limited. A Cochrane review [18] identified four trials with at least a six-month follow-up, reported in five papers [19-23]. Two of these trials evaluated a combined internet and mobile phone programme and showed evidence of effectiveness at 12 months [22,23]. The trials of text messaging showed positive short-term results but no evidence of longer-term effectiveness. Since that review was conducted, a large trial of the txt2stop intervention in the UK has been published, showing a positive effect on biochemically verified continuous abstinence at six months [24]. The intervention was also cost-effective [25].

The system we have developed differs from txt2stop in that the text messages in the iQuit system are highly tailored to the individual smoker using both information collected at baseline via an online questionnaire and information obtained subsequently from the smoker via two-way texting (sometimes referred to as dynamic tailoring). Unlike previous trials, iQuit in Practice will evaluate an intervention in which tailored text messaging is used to support smoking cessation among smokers treated in primary care.

### Trial objectives

The objectives of the trial are:

1. To assess the *acceptability* of the intervention among the Smoking Cessation Advisers and the participants.

2. To assess the *feasibility* of the intervention and of aspects of the trial design and procedures.

3. To estimate the short-term *effectiveness* of the intervention in increasing the quit rate compared with usual care alone.

The findings will be used to modify the intervention and to inform the decision to proceed to a pragmatic trial to estimate longer-term effectiveness and cost-effectiveness.

## Methods/design

### Design of the study

The design is a two parallel group randomised controlled trial (RCT) with 1:1 individual allocation comparing usual care (Control) with usual care plus the iQuit system (Intervention).

### Participants

#### Inclusion/exclusion criteria

Patients are eligible for inclusion in the study if they meet all of the following criteria: current smoker (usually smokes at least one cigarette a day and has smoked in the seven days prior to randomisation date); able to read English and can provide written informed consent; wants to quit smoking and is willing to set a quit date within the 14 days after randomisation; aged 18 – 75 years; has a mobile phone and is familiar with sending and receiving SMS text messages; willing to participate in study and follow study procedures; not currently enrolled in another formal smoking cessation study or programme; not using nicotine replacement therapy (NRT), bupropion (Zyban), varenicline (Champix) or other pharmacotherapy at randomisation date.

Patients will be excluded if considered by their GP to be unsuitable for the project for any reason, for example people with severe mental impairment or severely or terminally ill. Patients with co-morbidities such as chronic obstructive pulmonary disease (COPD) or diabetes will not be excluded from the study unless their GP considers them unsuitable. In addition, a person living in the same household as an existing trial participant will not be excluded from participating in the trial if they meet the inclusion/exclusion criteria.

#### Recruitment: practices

Participants will be recruited from general practices in the East of England. Our previous studies [2,10] suggest that 20 is a feasible minimum target number of participating smokers per practice. Given the proposed sample size of 600 smokers, if 20 smokers were recruited from each practice, we would need 30 practices in total. However, some practices may be able and willing to recruit and treat more than 20 smokers, in which case the total number of participating practices will be fewer than 30, which would be desirable from a logistical standpoint.

To be eligible for the trial, a practice must have at least one SCA who has been trained to give level 2 smoking cessation advice and who has received refresher training within the last two years (or is willing to be trained to this level). The SCA(s) must have internet access from a computer in the consultation room and access to a printer. The practice should not be participating in any other smoking cessation research study. Preference will be given to practices that are geographically accessible from the study centre in Cambridge.

#### Recruitment: participants

Participants will be recruited by two methods:

1. Opportunistic recruitment. Patients who self-refer, or who are referred via another health professional, to the SCA for smoking cessation advice will be given (or sent via post) a Participant Information Sheet (PIS) and Participant Consent Form (PCF) by the practice reception staff. Posters will be displayed in the practice and study leaflets will be placed in the waiting area to encourage self-referral.

2. Proactive recruitment. Practices will be asked to identify potential participants from smoking status data collected in recent medical appointments. Recent smokers are likely to be current smokers. A list of potential participants will be generated from the practice database by a member of the primary care team. The list will then be pre-screened by a practice clinician for eligibility. A random sample of those eligible to participate will be selected and sent, via post from the GP Practice, a covering letter from patient’s GP providing information as to why they are being written to (‘smoker’ status on practice database), details of smoking cessation advice available to them, a leaflet outlining the study, and a copy of the PIS and PCF.

The consent procedure will follow Good Clinical Practice guidelines [26]. If they have not already done so, those interested in participating in the research will be requested to phone or attend the practice to make an appointment with the SCA. When potential participants attend their initial appointment with the SCA, they should already have received information regarding the trial, as outlined above. This will ensure that participants have had at least 24 hours to consider whether to take part in the study. They will be asked whether they wish to take part in the study, and their eligibility will be checked by the SCA. If they have not received information about the study prior to attending the consultation, they will be given an information pack by the SCA and invited to return at a later date.

Those who do not wish to participate, or who are ineligible, will receive usual care. Screening forms will be completed to record non-personal details of those individuals who are interested in taking part but found to be ineligible.

### Interventions

#### Control

Although there are some variations in practice, ‘usual care’ offered by SCAs for smoking cessation in the East of England typically consists of a brief discussion about smoking habits and history, measurement of expired-air carbon monoxide (CO), brief advice to quit (including the importance of setting a quit date), options for pharmacotherapy, a prescription, and arranging a follow-up visit or phone call. This is referred to as ‘level 2’ smoking cessation advice [27].

#### Intervention

The intervention consists of usual care, as described above, plus two components: a tailored advice report and a programme of tailored text messages:

#### (i) Advice report

The 30-item iQuit online questionnaire includes questions on the following: demographic factors; smoking habits and history, including cigarette consumption and length of longest previous abstinence; motivation and determination to quit; reasons for quitting; dependence; self-image; pros and cons of quitting; difficult situations; children; living with other smokers; social support; and current health problems. During the consultation, the SCA will ask the participant the questions and enter their answers on the online questionnaire. If the participant is allocated to the intervention condition, the program will generate an advice report on completion of the questionnaire. The SCA will print out the report and give it to the smoker. The report, which is approximately three A4 pages in length, contains detailed advice on quitting that is highly tailored to the individual smoker using their responses to the questionnaire. The content of the report is based on: relevant theories of smoking cessation and behaviour change, including social cognitive theory [28] and the perspectives on change model [29]; the findings from a previous study of predictors of quit attempts and success in a sample of callers to the Quitline; input from the Quitline counsellors; and ‘received wisdom’ about smoking cessation (e.g. the importance of setting a quit date). The program can generate over 3300 million different reports.

#### (ii) Text messaging

This is a 90-day program of automated text messages sent to the smoker’s mobile phone. The messages will start the day before the quit date that the smoker sets during the consultation with the SCA. The number of messages sent each day will be 0, 1 or 2. In the later part of the program, fewer messages will be sent (i.e. 0 messages will be sent on a greater proportion of days). The messages are designed to remind smokers about their quit attempt, to provide information about reasons for quitting, to provide general encouragement, to increase and maintain motivation to quit, to boost confidence and to remind smokers of difficult situations and how to cope with them.

A key feature of the system, which distinguishes it from other texting systems for smoking cessation, is that the messages are individually tailored using both the information collected on the online questionnaire and additional information obtained from the smoker during their quit attempt about their smoking status. To obtain the latter information, query messages will be sent to the participant on two occasions during the 90-day period, to which they can text either YES or NO. In addition to the scheduled supportive and query messages, the smoker will also be able to text HELP or SLIP to immediately receive a message designed to provide support if he/she is tempted to smoke (HELP) or has just had a lapse (SLIP). The smoker can text STOP to stop receiving any further text messages.

The web-based program and advice report were piloted in five general practices in Cambridgeshire. The nurses were positive about the computer-based assessment and printed report which they felt aided consultation and engagement with the smokers. Although using the program increased the length of the consultation, the nurses thought that it integrated well with the usual procedures and said that they would continue to use the program if it were available. The text messages have been developed following extensive qualitative work with smokers, including focus groups and individual interviews [17,30].

#### Training

SCAs will receive training on how to deliver the intervention and the importance of collecting and recording accurate and complete data from all participants. Training will also include other research skills including the taking of informed consent and the issue of confidentiality within a research setting. In a one-to-one session with one of the research team, designed to rehearse what will happen in the consultation with the smoker, SCAs will be trained to use the iQuit computer program by completing the online questionnaire, to follow the research protocol, to complete case report forms, and to use a Smokerlyser (Bedfont) CO monitor and an audio recorder. Follow-up training and ongoing support will also be provided by the research team.

### Procedure: initial consultation

Once the patient has consented to participate in the study, the SCA will provide brief smoking cessation advice, as per usual practice. The participant is required to set a quit date within the next 14 days. Expired-air CO level will also be measured. The CO monitors will be calibrated regularly by the study team.

During the consultation, the SCA will log on to the web-based iQuit program via the practice computer using a personal SCA identification number. The SCA will be prompted to enter answers to the first set of questions (Part 1) on the online questionnaire, as they complete it in consultation with the participant. These questions, which will be completed for all participants in the study, include gender, age, cigarette consumption, longest period of abstinence, strength of motivation and determination to quit smoking, and quit date (which must be in the next 14 days). The SCA will also enter the participant’s CO level on the questionnaire. This information will be used to characterise the sample and to check the baseline comparability of the intervention and control groups.

For those participants randomised to the Control group, only the first set of questions on the online questionnaire will be completed and no advice report will be generated. For those participants randomised to the Intervention group, the SCA will ask a second set of questions (Part 2) and will enter the answers on the online questionnaire. The 17 questions in Part 2 include whether participants think of themselves as addicted to smoking, most important reason for quitting, most difficult situation in which to resist smoking, main advantage and main disadvantage of quitting, whether they live with other adults who smoke, whether they have any health problems linked with smoking, and their mobile phone number. The answers to both sets of questions (Part 1 and Part 2) will be used to generate the tailored advice report. The SCA prints out the report and gives it to the smoker and then concludes their routine support by arranging a prescription for NRT or other pharmacological treatment, if appropriate, and arranging a routine NHS follow-up visit four weeks from the quit date. Some practices may also conduct interim follow-up visits according to their standard practice.

During the baseline and four-week follow-up consultations, the SCA will manually complete case report forms (CRFs). These will record data collected by the NHS routinely for smoking cessation targets (including ethnic group, occupation code, smoking cessation medications prescribed, outcome) to allow comparison of study quit rates with NHS reported rates. The SCA will manually record the time at start and completion of the CRFs, and the iQuit program will record the time taken to complete the first and second set of questions on the online questionnaire.

Participants randomised to the Intervention group will be sent tailored text messages from a web server at the study centre over a three-month period, starting the day before their quit date. To cover any costs associated with receiving and sending text messages for the study, intervention participants will be sent a £5 voucher, posted out with the eight-week follow-up questionnaire. Control participants will not be sent any text messages.

#### Audiotaping of consultations

SCAs will be asked to audiotape a sample of intervention and control consultations. The tapes will be used to assess the fidelity of the intervention and to describe the content of ‘standard advice’. They will also provide data on the time taken to deliver the intervention and standard advice.

### Randomisation

Participating smokers will be randomised by the iQuit computer program during the consultation such that a particular SCA will see approximately equal numbers of intervention and control participants i.e. individual level randomisation stratified by SCA. The sequence will be generated by a computer-based random number generator using the method of random permuted blocks with block sizes of four and six to make the sequence difficult to predict without leading to a major imbalance in numbers between intervention and control groups if a block is incomplete at the end of recruitment. The allocation sequence will be stored on the web server database prior to the trial start. It will be accessible to the investigators but not to the SCAs or participants. Allocation will be made by the trial web server as soon as Part 1 of the questionnaire has been submitted. At this point, the SCA and the participant will become unblinded to group allocation.

### Procedure: follow-up

All participants will have a routine NHS follow-up at four weeks from quit date and two research follow-ups, at eight weeks and six months from randomisation date.

#### 4-week follow-up

Before they leave their initial appointment with the SCA, participants will be asked to make a follow-up appointment. This visit will occur approximately four weeks after the participant’s quit date (-3 days or +14 days), as per usual NHS practice for smoking cessation. The SCA will record smoking status and other data collected by the NHS routinely for smoking cessation targets. CO level will also be measured. The SCA will also check contact details for the eight-week and six-month follow-ups.

In some instances, practices may follow-up patients by telephone or by postal questionnaire. The research team will collect data on the type of follow-up conducted at four weeks and any interim follow-ups conducted. If participants do not attend the practice for their scheduled follow-up appointment, SCAs will be encouraged to contact them and invite them to attend on an alternative date. Participants who do not attend a four-week follow-up appointment with the SCA at the practice will still be included in the study for follow-up at eight weeks and six months.

#### 8-week follow-up

The eight-week follow-up will be via a postal questionnaire sent directly to participants from the study centre. The questionnaire will assess self-reported abstinence and the acceptability and feasibility of the intervention by those participants who received it. Non-responders (those whose questionnaire has not been returned to the study centre within 2 weeks) will be phoned by a research interviewer, initially blinded to the participant’s group allocation (control or intervention). The participant will be given the option of completing the questionnaire over the phone or completing and returning it by post (they will be sent another copy if they need one). For the first option, if the participant is unwilling to complete the full questionnaire over the phone, only the smoking outcome questions will be asked. If it is not possible to contact the participant by telephone after six attempts, no further attempt will be made to collect eight-week follow-up data from that individual; however, they will still be included in the study for follow-up at six months.

#### 6-month follow-up

The same procedure will be used for the six-month follow-up. If it is not possible to contact the participant after six attempts, he/she will be recorded as lost to follow-up.

### Questionnaire survey and interviews with intervention deliverers

In order to assess the acceptability of the iQuit intervention for those delivering the intervention and to assess the feasibility of the intervention and aspects of the trial design, SCAs will be invited to complete a postal questionnaire, providing feedback on the programme including research elements such as practice support, audio recording and managing paperwork. A sub-sample of SCAs, consisting of nurses and HCAs from high and low recruiting practices will be purposively sampled to participate in semi-structured interviews to canvass their views on participating in the study.

Interviews will be completed at the practice, last up to one hour and be audio recorded and transcribed verbatim.

### Outcome measures

#### Effectiveness

(i) Primary outcome measure

The primary outcome measure of effectiveness will be self-report of being abstinent from smoking for at least two weeks at eight-week follow-up from randomisation date, as assessed by postal questionnaire or telephone interview.

(ii) Secondary outcome measures

Secondary outcome measures will include:

(a) CO-verified self-report of being abstinent from smoking at four-week follow-up from quit date for at least two weeks, assessed at an appointment with the SCA. (We will use the definition recommended for use by local Stop Smoking Services in England [31]: a CO reading that is assessed 25 to 42 days from quit date and is less than 10 parts per million.)

(b) Self-reported prolonged abstinence (at least 3 months) at six-month follow-up from randomisation date, assessed by postal questionnaire or telephone interview. Because follow up will be conducted by post and/or telephone call with a research interviewer, we do not plan to validate self-reports at six months [32]. Furthermore, it would be difficult to bring participants into the surgery for an additional CO measure at this time point. The main alternative, obtaining saliva samples by post for cotinine assay, has several disadvantages: it may yield a low response rate; measuring cotinine at one time point cannot validate prolonged abstinence; smoking behaviour may change between the questionnaire and receipt of the saliva kit; it is impossible to check whether a returned saliva sample is from a particular participant – samples may be substituted; and a significant proportion of participants may still be using nicotine replacement at follow up.

#### Feasibility

Measures for assessing feasibility will include: the proportion of practices approached who agree to participate; the proportion of smokers who respond to the proactive recruitment letter; the number of smokers per practice recruited into the study in a six-month period; response rates to postal follow-up questionnaires; increase in length of consultation.

#### Acceptability

(i) To the SCAs

SCAs’ evaluation of using the iQuit program, as assessed by postal questionnaire (how easy was it to use; how helpful they found it; how it could be improved; would they continue to use it if it were available).

(ii) To the participants (intervention group only)

(a) Participants’ evaluation of the printed advice report, as assessed by postal questionnaire at eight weeks (whether they kept the report; how much of it they read; how clear, useful and relevant the advice was; what they particularly liked and disliked about the report).

(a) Participants’ evaluation of the text messaging component, as assessed by postal questionnaires at eight weeks and six months (e.g. how useful and relevant they found the messages; whether there were too many or not enough; whether they covered the right topics; how the system could be improved).

(a) Proportions of participants who used the HELP and SLIP features; frequency of use.

(a) Proportion of participants who texted STOP to stop receiving further messages; when they did this (number of days into the program); why they did this (assessed on 6-month follow-up questionnaire).

### Sample size, power and precision

This is a proof-of-concept trial designed to yield information about the acceptability, feasibility and short-term effectiveness of the intervention, but not to give a definitive answer to the question of whether the intervention is effective in the longer term.

For effectiveness, we have chosen to power the study on outcome at eight weeks (self-reported abstinence for at least 2 weeks at 8 week follow-up from randomisation date). A sample size of 300 per group would give 80% power to detect an increase in quit rate from 20% to 30% (alpha = 0.05, 2-sided test).

The six-month outcome will provide valuable additional information. For comparison between groups at six-month follow-up, a 95% confidence interval will be calculated to estimate the precision of the estimate of the difference in quit rates. Assuming a six-month quit rate of 8% in the control group, based on pooled rates observed in similar control groups reported in a Cochrane review of self-help [7], an observed increase of 5% in the six-month quit rate from 8% to 13% (RR 1.65) would be estimated with an accompanying 95% confidence interval having a width of +/-5%, given the trial size of 600. Such an observed increase would be borderline statistically significant, though the trial is limited to having 51% power to detect a true underlying difference of 5% as statistically significant and a larger trial would be needed to distinguish reliably a true increase from a Type 1 error. Importantly, if a 5% increase is observed, then an underlying population relative risk of 1.5 or higher will be estimated to exist with posterior probability 74%, using a Bayesian statistical approach where no prior information is added to that obtained through the trial. This probability will inform the decision of whether to proceed to a larger, pragmatic trial; the 95% confidence interval would then be used to inform the choice of a plausible and worthwhile effect size from which to power this trial with a primary outcome of six-month or 12-month quit rate.

### Statistical analysis

An analysis plan will be prepared by the study statistician prior to analysis. The two groups will be compared using chi-squared tests and logistic regression analysis for binary outcome measures, and independent t-tests, analysis of variance and linear regression analysis for continuous measures. All tests will be two-sided using an alpha of 0.05.

The main analyses of quit rates will be conducted on an intention-to-treat basis, making the usual assumption that participants lost to follow up are smoking, although we will also conduct sensitivity analyses using a range of less severe assumptions informed by available baseline and interim data [33]. A further sensitivity analysis will involve estimating the intervention effect allowing for clustering of participant outcomes by household. As the mean cluster size (participants per household) will be close to 1, the results and conclusions are not expected to be materially different from the main analysis. The intra-household correlation coefficient will be estimated in order to inform the design and sample size of any subsequent trial.

Proportions, such as many of the secondary outcomes of acceptability and feasibility, and loss to follow up, will be reported overall and for each arm and compared between arms with a p-value from Fisher’s exact test and a 95% confidence interval calculated by the Clopper-Pearson method. Proportions that are defined in a single arm will be estimated with an exact 95% confidence interval using the binomial distribution.

Additional analyses will include an examination of potential effect modifiers (i.e. was the intervention more effective in particular subgroups of smokers?), predictors of quit attempts and success in quitting, and a comparison of study quit rates with NHS reported rates. The cost of delivering the intervention will be assessed in terms of the average length of the consultation in the two conditions.

### Ethical approval

Ethical approval for the trial was granted by Cambridgeshire 2 Research Ethics Committee (REC reference number: 09/H0308/87).

## Discussion

The objectives of this trial are to assess acceptability, feasibility and short-term effectiveness of the iQuit in Practice intervention. The intervention needs to be acceptable to the SCAs who deliver it (the tailored advice report) and to the participants who receive it (the tailored advice report and the tailored text messages). Acceptability to the SCAs will be assessed by both postal questionnaires and semi-structured interviews. These will also address aspects of feasibility, along with measures such as the number of smokers per practice recruited into the study in a six-month period. Acceptability to the participants will be assessed by postal questionnaires and by measures of how they engage with the text messaging programme, for example the proportion who opt to stop receiving text messages.

With regard to effectiveness, we designed the trial to maximise both internal and external validity. The participant will be randomised by computer program during the consultation. The allocation sequence will not be accessible to the SCAs or to the participants. However, as soon as the participant has been randomised, both the SCA and the participant will know which group the participant has been allocated to. If the participant is allocated to the intervention group, the SCA will ask the participant more questions and enter the answers on the online questionnaire. They will then print out the tailored advice report and give it to the participant. The content of the remainder of the consultation should be identical for intervention and control participants. However, given that both SCA and participant will become unblinded during the consultation, it is possible that there will be systematic differences between intervention and control conditions in the usual care component in the later part of the consultation; for example, SCAs may unwittingly or deliberately provide additional advice to control participants to compensate for them having been allocated to this condition, or control participants may ask what advice they would have received had they been allocated to the intervention group. Such effects may dilute differences between groups. A sample of intervention and control consultations will be audiotaped so that the content of the consultations can be compared, which may allow us to detect (though not prevent) this possible source of performance bias.

Outcome assessment will be by postal questionnaire or by telephone interview conducted by a research interviewer. The interviewer will initially be blinded to the participant’s group allocation, and the primary outcome will be assessed at the start of the interview, but the interviewer will become unblinded during the interview when they ask about the intervention. Whether the primary outcome is collected by questionnaire or interview, there is a risk of bias when the outcome is based on self-report and participants know their group allocation. Use of an objective measure would in principle eliminate outcome assessment bias. We carefully considered whether to try to obtain saliva samples by post for cotinine assay, but decided against this because it has several disadvantages; for example, it may yield a low response rate.

In this trial, external validity is increased by using health professionals (practice nurses and health care assistants) rather than research staff to deliver the intervention (tailored advice report) and the control intervention (usual care) in a general practice setting and by employing minimal exclusion criteria for both practices and patients. The tailored advice report component of the intervention was designed to be easily integrated into a usual care consultation and the text messaging component to be delivered independently of the general practice setting.

The findings of the trial will be used to refine the intervention and to inform the decision to proceed to a pragmatic trial of effectiveness and cost-effectiveness. Such a trial would include a more detailed assessment of costs, and the primary outcome for effectiveness would be assessed at 6 or 12 months. We would consider using a cluster randomised design (with practice as the cluster) instead of individual randomisation, and we would attempt to minimise the time that the SCAs spend on research procedures in the consultation and to reduce the measurement burden for participants and SCAs.

## Competing interests

The authors declare that they have no competing interests.

## Authors’ contributions

SS is chief investigator and grant holder, conceived of the study, contributed to its design and led on writing the manuscript; SSm and FN co-ordinated the trial and helped to draft the manuscript; JB designed the trial database; DM translated the tailoring mechanism into a web-based application and programmed the online questionnaire; AP is the trial statistician and advised on design and statistical analysis and developed the statistical analysis plan; JJ led the development and piloting of the text messaging component of the intervention, developed study materials, facilitated the training of SCAs and is responsible for day-to-day management of the trial; SB developed study materials, recruited and liaised with practice teams and trained SCAs in intervention delivery; MS led on the participant telephone follow-up interviews; HG led the development of the advice report component of the intervention and advised on the design of the trial and the measures. All authors read and approved the final manuscript.

## Pre-publication history

The pre-publication history for this paper can be accessed here:

http://www.biomedcentral.com/1471-2458/13/324/prepub
